# Uncovering Gaps in Knowledge: A Survey of Belgian General Practitioners’ Awareness of Legionnaires’ Disease Diagnostic Testing

**DOI:** 10.3390/idr16050063

**Published:** 2024-08-27

**Authors:** Marco Moretti, Julien Van Nedervelde, Robin Vanstokstraeten, Lucie Seyler, Fedoua Echahidi, Benoit Prevost, Delphine Martiny, Ingrid Wybo, Charlotte Michel

**Affiliations:** 1 Department of Internal Medicine and Infectious Diseases, Universitair Ziekenhuis Brussel (UZ Brussel), Vrije Universiteit Brussel (VUB), 1090 Brussels, Belgium; lucie.seyler@uzbrussel.be; 2 European Study Group for Legionella Infections (ESGLI), 4051 Basel, Switzerland; fedoua.echahidi@uzbrussel.be (F.E.); ingrid.wybo@uzbrussel.be (I.W.); charlotte.michel@uzbrussel.be (C.M.); 3 Independent Researcher in Primary Care, 6222 Villers-la-Ville, Belgium; docteur.julienvn@gmail.com; 4 Department of Microbiology and Infection Control, National Reference Centre for Legionella pneumophila, Universitair Ziekenhuis Brussel (UZ Brussel), Vrije Universiteit Brussel (VUB), 1090 Brussels, Belgium; robin.vanstokstraeten@uzbrussel.be; 5 National Reference Centre for Legionella pneumophila, Laboratoires des Hôpitaux Universitaires de Bruxelles—Universitair Laboratorium Brussel (LHUB—ULB), 1000 Brussels, Belgium; benoit.prevost@lhub-ulb.be (B.P.); delphine.martiny@lhub-ulb.be (D.M.)

**Keywords:** *Legionella* infection, primary care medicine, *Legionella pneumophila*, diagnostic tools, survey study

## Abstract

***Background*:** The incidence of Legionnaires’ disease (LD) is increasing steadily in Europe. Its early diagnosis by general practitioners (GPs) is crucial for better patient outcomes. ***Study objectives*:** This study assessed Belgian GPs’ knowledge about LD and the accessibility of diagnostic tests in their practices. ***Methods*:** A specifically designed questionnaire was distributed to actively practicing GPs, including primary care trainees, between 31 January 2022 and 13 March 2022. This survey targeted approximately 4200 GPs with an estimated population catchment of 30% of the actively working Belgian GPs. ***Results*:** The response rate was estimated at 3%. Over 70% of the GPs correctly identified the LD occurrence peak, major risk factors, and clinical manifestations. While 62% of participants preferred the *Legionella pneumophila* urinary antigen test (UAT) as a primary diagnostic method, 75% were unsure about its availability within their laboratories and 82% had not prescribed it in the last year. Finally, 76% expressed a desire for additional information on this topic. ***Conclusions*:** Belgian GPs should evaluate the possibility of conducting UAT testing in their laboratories to enhance LD case management and improve their preparedness. Furthermore, initiatives should be implemented to improve communication between specialists and GPs and develop educational programs directed at Belgian GPs.

## 1. Introduction

Legionnaires’ disease (LD) is a type of pneumonia predominantly caused by *Legionella pneumophila* serogroup 1 (LpS1), which is responsible for 82% of cases worldwide [1]. The epidemiological data from Europe reveal a consistent rise in LD cases over the last decade [1]. Similar trends were observed in Belgium, with incidences of LD of 3.1 and 3.2/100.000 inhabitants in 2021 and 2022, respectively (Supplementary Materials Figure S1) [2,3,4]. Although the peak prevalence of LD usually occurs during the summer months, cases can be encountered all year-round [2,3,4,5]. The diagnosis of LD is most commonly performed using the *Legionella pneumophila* urinary antigen test (UAT) in clinical microbiology laboratories (CMLs) due to its ease of performance and rapid results [6]. However, commercially available UAT kits primarily target LpS1 and show a decreased sensitivity for other serogroups [1,2]. The reported mortality rate from LD in Belgium reached 14% in 2022 [2,4], but prompt diagnosis and treatment have been clearly linked to improved outcomes in both sporadic and outbreak situations [7,8]. While LD cases usually occur outside outbreak settings [2,3,4], several clusters have been reported in Belgium over the years. One of them was a major LD outbreak in 2016 with a cooling tower as the outbreak source [9]. The epidemiological investigation revealed several weaknesses in the management of those cases, among which were diagnostic delays. The median time between symptom onset and case confirmation was as long as 30 days. As general practitioners (GPs) are responsible for case confirmation by using the most appropriate diagnostic tests, GPs play a crucial role in such rural outbreaks [10,11]. In addition to diagnostic delays, underreporting and, more importantly, underdiagnosis of LD are other issues [4,12].

As LD cases are expected to rise further in Belgium, so will cases seen by Belgian GPs. However, GPs’ level of knowledge and understanding of this severe infection remain unclear, in particular regarding diagnostic procedures and access to diagnostic testing within their practice. Previous studies have suggested a lack of GP awareness of LD, even following outbreaks in other countries [10,11].

This study aimed to evaluate Belgian GPs’ familiarity with LD using a questionnaire. The survey assessed their current knowledge of the subject, their management of suspected LD cases, and the accessibility of diagnostic tests within their practices.

## 2. Materials and Methods

### 2.1. Study Design

This study is a cross-sectional survey and was elaborated following the Reporting Guidelines for Survey Research [13].

### 2.2. Research Tool

A self-administered questionnaire was developed in both French and Dutch specifically for this study, as existing surveys did not address our research objectives. The questionnaire was not formally validated before use. It does not employ any scoring procedures and is divided into six sections: Demographics, LD Experience, LD Knowledge, CML Services, Treatment, and Additional Information. The questionnaire contains 24 questions with 19 multiple-choice and five open-ended questions requiring brief responses (Supplementary Materials Table S1).

### 2.3. Sample Selection

The target population was general practitioners (GPs) actively practicing in all three regions of Belgium: Wallonia, Flanders, and Brussels-Capital. This included primary care trainees. Participation was voluntary and anonymous to ensure confidentiality. No a priori pre-sample size calculation was conducted, aiming to reach most of the GPs working on the Belgian territory.

### 2.4. Survey Administration

**Figure 1:**
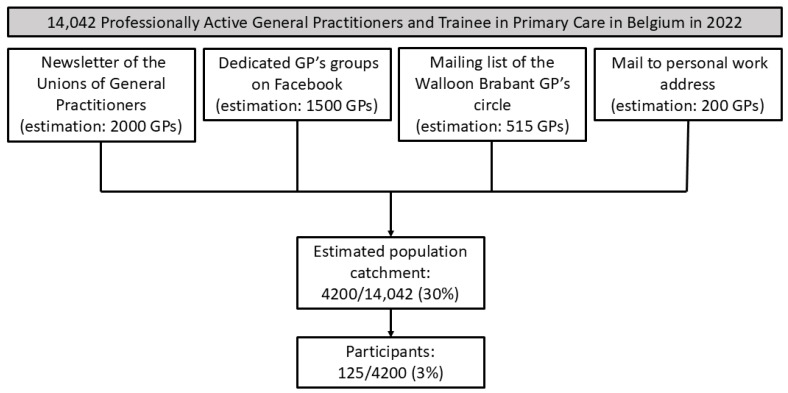
Study flowchart.

The survey was distributed between 31 January 2022 and 13 March 2022. Figure 1 illustrates the distribution channels used to reach participating GPs. The present survey was not designed to account for a response rate for each distribution method individually. Instead, we looked at the global response rate. The questionnaire was available online through Google Forms, a survey platform developed by Google.

### 2.5. Statistical Methods

Data were presented as median and interquartile range for continuous variables, and as numbers and proportions for categorical variables. Nonresponse bias was addressed by estimating the response rate and evaluating the first four questions of the survey, which aimed to assess the participants’ representativeness. Analyses were performed with IBM SPSS Statistics for Windows, Version 20.0, Armonk, NY, USA: IBM Corp., 2011.

## 3. Results

### 3.1. Response Rate and Nonresponse Bias

Considering the number of professionally active GPs at the time, the estimated population catchment of the survey was 30% (4.200/14,042) of all the Belgian GPs. One hundred and twenty-five of them completed the questionnaires. Therefore, the estimated response rate was 3% (125/4200) (Figure 1).

The majority of the respondents were trainees at 28% (n = 35), practicing in the Walloon Brabant province at 27% (n = 34), working within a GP association at 37% (n = 42), and practicing within an urban area at 45% (n = 56). Overall, 78% of the participants (n = 97) had never diagnosed a patient with LD. Table 1 illustrates the demographics of the participants.

### 3.2. Knowledge on Legionnaires’ Disease

The large majority of respondents (n = 97; 78%) considered LD occurring particularly in the summer. The most important patients’ history-taking elements were a link with water systems in the professional setting (n = 119; 95%) or the use of a swimming pool, jacuzzi, or spa (n = 104; 83%), being immunocompromised, or having chronic lung diseases. Signs of pneumonia at auscultation were considered the most common clinical findings (n = 115; 92%), and the large majority found radiological confirmation as meaningful (n = 94; 75%). The preferred diagnostic tool was UAT for 62% of respondents, followed by specific molecular testing on respiratory samples (18%). Globally, we observed an increasing trend towards using serology as the preferred diagnostic tool for LD with increasing years of experience as a GP. Among primary care trainees, 77% favored UAT, and 3% preferred serology. Among the most experienced GPs (>40 years of experience), 40% and 33% considered UAT and serology, respectively, as the reference test for LD diagnosis. Also, 66% of trainees identified LpS1 as the predominant *Legionella* serogroup detected by UAT, and this proportion tended to drop with increasing years of practice. Ten percent of GPs with 30–39 years of experience perceived LpS1 as the main serogroup identified by UAT. Finally, if a strong clinical suspicion persisted after negative UAT, 30% (n = 38) of participants said they would contact a specialist physician, while 13% (n = 16) would ask for serological testing, and 12% (n = 15) would request a PCR on a respiratory sample. Supplementary Materials Table S2 summarizes the general knowledge about LD among GPs based on their survey responses; global data are provided along with replies divided into years-of-experience categories.

### 3.3. Diagnostic Tool Availability in Legionnaires’ Disease

**Figure 2:**
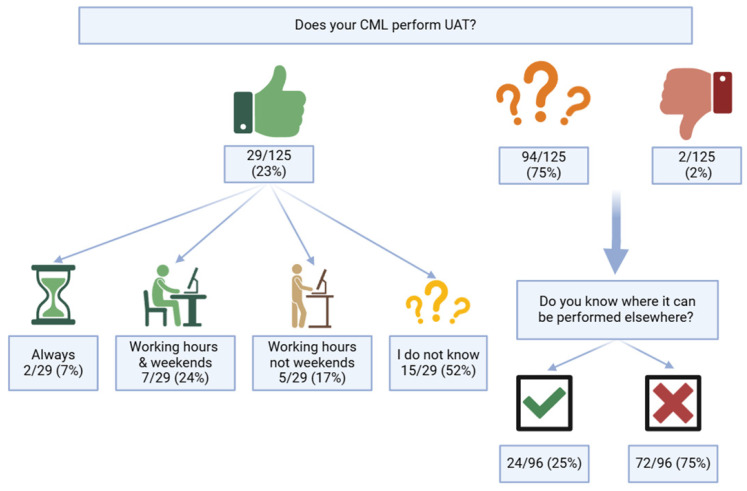
Availability of *Legionella pneumophila* urinary antigen test in primary care medicine; CML: clinical microbiology laboratory; UAT: *Legionella pneumophila* urinary antigen test.

Seventy-five percent of respondents (n = 94) did not know if the CML they worked with performed UAT. Furthermore, of the 29 GPs who did know, only 2 reported guaranteed 24/7 access to UAT testing. Also, 82% of the respondents (n = 94) had never prescribed UAT in the preceding year, and the remaining 18% prescribed it less than 20 times in the last year. Figure 2 and Supplementary Materials Table S3 summarize the answers of the GPs on the availability of diagnostic tools in their daily practice.

### 3.4. Antibiotic Treatment in Legionnaires’ Disease

Azithromycin was the preferred antibiotic for the treatment of LD (n = 38; 31%), followed by clarithromycin (n = 30; 24%) and levofloxacin (n = 29; 23%). The median duration of treatment was 10 days, with an interquartile range (IQR) of 7 to 14. A trend towards prescribing longer antimicrobial courses was observed in the more experienced GPs categories. Primary care trainees reported a median duration of treatment of 7 days (IQR: 5–10), whereas GPs with the most experience recommended a median treatment duration of 10 days (IQR: 7–15). Notably, 14% of all respondents (n = 17) replied that they did not know the recommended length of therapy. Supplementary Materials Table S4 illustrates the GPs’ responses regarding treatment modalities.

### 3.5. Additional Information

Additionally, 98% of participants (n = 123) were not familiar with the Belgian National Reference Center for *Legionella pneumophila* (NRC), and 76% (n = 95) would like to receive more training and better access to information on LD.

## 4. Discussion

### 4.1. Summary of Key Findings

The current survey involved approximately 22% of the practicing general practitioners (GPs) in Belgium, and resulted in a response rate of 3%. Overall, the surveyed GPs displayed a satisfactory understanding of Legionnaires’ disease (LD) occurrence, associated risk factors, and clinical manifestations. Considering treatment options, most GPs aligned with guideline recommendations [14]. However, despite expressing a preference for urinary antigen tests (UATs) as their primary diagnostic method, 75% were uncertain if their respective laboratories conducted UATs. This uncertainty is further reflected by the low utilization rate of UATs, with 82% of participants reporting no UAT prescriptions in the previous year. Furthermore, if UAT results were negative but clinical suspicion remained, only 12% of GPs would request a molecular test, and 6% a culture on a respiratory specimen. The infrequent use of UATs and uncertainty about LD diagnostics suggest a knowledge gap among Belgian GPs. Additionally, a significant proportion of GPs demonstrated unfamiliarity with the *Legionella* National Reference Center (NRC), with 76% expressing interest in further information.

### 4.2. Interpretation of Study Findings

The UAT is the cornerstone of LD diagnosis, accounting for 69% of confirmed cases in Belgium. Potential limitations in the utilization of UATs by Belgian GPs could be attributed to strict reimbursement rules. The reimbursement of a UAT in Belgium has only been granted since 2016 in cases of pneumonia in hospitalized patients. However, a prompt diagnosis is crucial to reduce mortality and enhance outbreak management [7,8,9]. A retrospective study found an association between higher case fatality rates and the use of diagnostic methods other than the UAT [8]. Therefore, GPs should inform themselves about the availability of UATs in their CML to improve their access to this quick diagnostic tool. We believe that an optimized use of UATs by Belgian GPs could improve patients’ outcomes and expedite case recognition during rural outbreaks.

It is crucial to be aware that a UAT can produce negative results in up to 12% of cases, particularly with infections by non-LpS1 strains [1,2,4]. Molecular diagnostics is gaining recognition as a central tool, especially in cases with negative UAT results, and as a valuable complement to traditional *Legionella* cultures. While broncho-alveolar lavage remains the recommended specimen for these tests, growing evidence supports their use on less invasive samples like sputum [15,16]. Cultures and nucleic acid amplification tests should both be requested in cases of clinical suspicion of LD and negative UAT results. This comprehensive approach ensures a higher likelihood of accurate diagnosis, which is crucial for an improvement in outcomes. A similar survey conducted in Japan in 2021 revealed that most participating GPs did not prescribe additional tests for the diagnosis of LD when a UAT was negative. One of the main reasons for this was the unavailability of further testing in their local CML [17]. Therefore, Belgian GPs should be aware of the diagnostic tests offered in their CML, and, in case of persistent suspicion and unavailability of testing, consider referring to specialists. Finally, *Legionella* serological testing is not routinely used to diagnose acute LD due to a lack of specificity. Serological diagnosis necessitates paired samples collected four weeks apart, rendering it unsuitable for the time-sensitive needs of acute cases. Its use is primarily restricted to epidemiological investigations during outbreaks [18].

The guidelines for the treatment of LD are currently based on evidence primarily derived from observational studies [14,19,20]. Macrolides, particularly azithromycin, and fluoroquinolones, such as levofloxacin, constitute the mainstay of LD therapy. There is no evidence suggesting the superiority of one class over the other, and both can be used as monotherapy [14,21]. In cases of uncomplicated, community-acquired pneumonia, a short antibiotic course may be considered: 500 mg azithromycin once daily for 3 to 5 days, or 750 mg levofloxacin once daily for 5 days [14]. The available evidence does not support the use of combination therapy (macrolides and fluoroquinolones) in non-critically ill patients, which may even increase the risk of adverse reactions [14,21]. Prompt administration of effective therapy is associated with a reduction in mortality, further stressing the need for quick access and efficient microbiological diagnostic tools [7,8,14].

This study also revealed a concerning lack of familiarity among GPs with the *Belgian* National Reference Center for *Legionella pneumophila* (NRC). In practice, this laboratory is more a reference for clinical microbiologists, but effective outbreak management requires strong collaboration between GPs, peripheral CMLs, public health authorities, and the NRC. Continuous education is crucial to improve the management of both outbreaks and sporadic cases. Additionally, initiatives should be implemented to improve communication channels between specialists and GPs and develop educational programs for Belgian GPs.

### 4.3. Study Strengths and Limitations

The strengths of this study lie in its originality. To our knowledge, this is the first survey in Belgium focusing on the knowledge and awareness of LD among local GPs. The results of the present study might promote an improvement in LD diagnosis in Belgium and ameliorate patients’ outcomes.

Several limitations must be acknowledged. The low response rate might not eliminate confounding factors sufficiently. Therefore, the study may not be representative of the Belgian GPs nationally. For instance, most respondents were from the two French-speaking regions of Belgium and had their practice within a city. This could be the result of the distribution channels through which we chose to carry out this study. Finally, nationwide studies are requested to augment GPs’ participation and increase their awareness of this emerging disease. Those are needed before the generalization of these study results.

## 5. Conclusions

The present survey uncovered a knowledge gap among Belgian GPs regarding the diagnostic tests for LD. Even though most respondents identified the UAT as the preferred diagnostic test in suspected cases of LD, three-quarters of them were unaware whether their CML could perform this test. Furthermore, over 80% of the surveyed GPs had not requested a UAT in the last year. Notably, 76% of the respondents were interested in supplementary education on this topic. Belgian GPs should evaluate the possibility of conducting UAT testing in their local laboratories to enhance LD case management and improve their preparedness. Initiatives should be taken to boost communication between specialists and GPs, and develop educational programs for Belgian GPs.

## Figures and Tables

**Table 1:** The participant’s demographics. GP: General practitioner.

**Years of experience** **Total n = 125**	Trainees *35 (28%)*	<10 years 26 (21%)		10–19 years *17 (14%)*		20–29 years *13 (10%)*		30–40 years 19 (15%)		>40 years 15 (12%)	
**Practice location** **Total n = 125**	Walloon Brabant *34 (27%)*	Brussel-capital *21 (17%)*	Hainaut *20 (16%)*	Namur *12 (10%)*	Liege *10 (8%)*	Flemish Brabant *8 (6%)*	West Flanders *6 (5%)*	Luxemburg *6 (5%)*	Antwerp *4 (3%)*	Limburg *2 (2%)*	East Flanders *2 (2%)*
**Type of practice** **Total n = 125**	GPs Association *42 (34%)*			Individual practice *34 (27%)*			Fee-for-service practice *30 (24%)*			Fixed-fee practice *19 (15%)*	
**Practicing area** **Total n = 125**	Urban *56 (45%)*			Semi-urban *44 (35%)*				Rural *25 (20%)*			

## Data Availability

The datasets used and/or analyzed during the current study are available from the corresponding author upon reasonable request.

## Supplementary Materials

The following supporting information can be downloaded at https://www.mdpi.com/article/10.3390/idr16050063/s1, Table S1: English rendition of the study’s survey, Table S2: Answers of the general practitioners about Legionnaires’ disease general knowledge, Table S3: Diagnostic tools availability for diagnosis of Legionnaire’s Disease in primary care medicine, Table S4: Answers of the general practitioners about Legionnaires’ disease favorite treatment; Figure S1: Belgian cases of Legionnaire’s disease confirmed by the National Reference Center for Legionella pneumophila (2011–2022).

## References

[1] European Centre for Disease Prevention and Control Legionnaires’ Disease—Annual Epidemiological Report for 2021. https://www.ecdc.europa.eu/en/publications-data/legionnaires-disease-annual-epidemiological-report-2021#:~:text=Stockholm%3A%20ECDC%3B%202023.&text=In%202021%2C%20the%20highest%20annual,cases%20per%20100%20000%20population.

[2] De Muylder G., Laisnez V., Verrnelen K., Echahidi F., Michel C., Martiny D., Hammami N., Van De Putte B., Kana C.C., Pierard D. (2022). Epidemiologische Surveillance van Legionellose in België. Sciensano, the Belgian National Epidemiology Reference. https://www.sciensano.be/sites/default/files/legionellose_2022_nl_2.pdf.

[3] Moretti M., Allard S.D., Dauby N., De Geyter D., Mahadeb B., Miendje V.Y., Balti E.V., Clevenbergh P. (2022). Clinical features of Legionnaires’ disease at three Belgian university hospitals, a retrospective study. Acta Clin. Belg..

[4] Echaihidi F., Prevost B., Martiny D., Wybo I., Piérard D., Michel C. Activity Report from 2011 to 2022 Reference Centre for Legionella Pneumophila UZ Brussel—LHUB-ULB. https://www.sciensano.be/sites/default/files/legionella_2011-2022_nrc_rapport_english_final.pdf.

[5] Buchholz U., Altmann D., Brodhun B. (2020). Differential Seasonality of Legionnaires’ Disease by Exposure Category. Int. J. Environ. Res. Public Health.

[6] El-Khatib Z., Richter L., Ressler J., Benka B. (2022). Diagnostic Study to Assess the Performance of a New Urinary Legionella Antigen Test-A National Study in Three Referral University Hospitals in Austria during 2014–2017. Int. J. Environ. Res. Public Health.

[7] Alvarez J., Domínguez A., Sabrià M., Ruiz L., Torner N., Cayla J., Barrabeig I., Sala M.R., Godoy P., Camps N. (2009). Impact of the Legionella urinary antigen test on epidemiological trends in community outbreaks of legionellosis in Catalonia, Spain, 1990–2004. Int. J. Infect. Dis..

[8] Dominguez A., Alvarez J., Sabria M., Carmona G., Torner N., Oviedo M., Cayla J., Minguell S., Barrabeig I., Sala M. (2009). Factors influencing the case-fatality rate of Legionnaires’ disease. Int. J. Tuberc. Lung Dis..

[9] Hammami N., Laisnez V., Wybo I., Uvijn D., Broucke C., Van Damme A., Van Zandweghe L., Bultynck W., Temmerman W., Van De Ginste L. (2019). A cluster of Legionnaires’ disease in Belgium linked to a cooling tower, August–September 2016: Practical approach and challenges. Epidemiol. Infect..

[10] Alaga K.C., Konja J.M., Salim A., Levine P., Smith S., Zervos M.J., Kilgore P. (2018). 675. Current Physician Knowledge, Attitudes, and Clinical Practice Regarding Legionnaires’ Disease in the Aftermath of the Flint Water Crisis in Genesee County, Michigan. Open Forum Infect. Dis.

[11] Kirrage D., Hunt D., Ibbotson S., McCloskey B., Reynolds G., Hawker J., Smith G.E., Olowokure B. (2007). Lessons learned from handling a large rural outbreak of Legionnaires’ disease: Hereford, UK 2003. Respir. Med..

[12] de Jong B., Hallström L.P. (2021). European Surveillance of Legionnaires’ Disease. Curr. Issues Mol. Biol..

[13] Bennett C., Khangura S., Brehaut J.C., Graham I.D., Moher D., Potter B.K., Grimshaw J.M. (2010). Reporting guidelines for survey research: An analysis of published guidance and reporting practices. PLoS Med..

[14] Cunha B.A., Burillo A., Bouza E. (2016). Legionnaires’ disease. Lancet.

[15] Moosavian M., Seyed-Mohammadi S., Saki M., Shahi F., Sima M.K., Afshahr D., Barati S. (2019). Loop-mediated isothermal amplification for detection of Legionella pneumophila in respiratory specimens of hospitalized patients in Ahvaz, southwest Iran. Infect. Drug Resist..

[16] Muyldermans A., Descheemaeker P., Boel A., Desmet S., Van Gasse N., Reynders M., on behalf of the National Expert Committee on Infectious Serology (2020). What is the risk of missing legionellosis relying on urinary antigen testing solely? A retrospective Belgian multicenter study. Eur. J. Clin. Microbiol. Infect. Dis..

[17] Kinjo T., Ito A., Ishii M., Komiya K., Yamasue M., Yamaguchi T., Imamura Y., Iwanaga N., Tateda K., Kawakami K. (2022). National survey of physicians in Japan regarding their use of diagnostic tests for legionellosis. J. Infect. Chemother..

[18] Centre for Disease Prevention and Control Legionella (Legionnaires’ Disease and Pontiac Fever)—Diagnosis, Treatment, and Prevention. https://www.cdc.gov/legionella/php/laboratories/?CDC_AAref_Val=https://www.cdc.gov/legionella/clinicians/diagnostic-testing.html.

[19] Kuzman I., Soldo I., Schönwald S., Čulig J. (1995). Azithromycin for treatment of community acquired pneumonia caused by Legionella pneumophila: A retrospective study. Scand. J. Infect. Dis..

[20] Yu V.L., Greenberg R.N., Zadeikis N., Stout J.E., Khashab M.M., Olson W.H., Tennenberg A.M. (2004). Levofloxacin Efficacy in the Treatment of Community-Acquired Legionellosis. Chest.

[21] Moretti M., De Boek L., Ilsen B., Demuyser T., Vanderhelst E. (2023). Therapeutical strategies in cavitary legionnaires’ disease, two cases from the field and a systematic review. Ann. Clin. Microbiol. Antimicrob..

