# Supplementary material for: Uncovering Gaps in Knowledge: A Survey of Belgian General Practitioners’ Awareness of Legionnaires’ Disease Diagnostic Testing
Source: Infect Dis Rep. 2024 Aug 27;16(5):820–7. doi: 10.3390/idr16050063 (PMC11417814; doi:10.3390/idr16050063)
Supplement: Supplementary file 1 [file idr-16-00063-s001.zip › idr-3118067-supplementary.pdf]

Supplementary Materials

Introduction Part

Supplementary Materials Figure S1 – Belgian cases of Legionnaire's disease confirmed by the National Reference Center for *Legionella pneumophila* (2011-2022). Figure shows constant increase of cases throughout the years with exception of 2020 and 2021. This is probably due to decrease tests capacity and quarantine measures taken to control COVID-19 pandemic. X-axis: time express in years; Y-axis: number of cases registered on the Belgian territory. CONF: confirmed case; PROB: probable case. F. Echaihidi *et al.* Activity report from 2011 to 2022. Accessed on the 24th of February 2024. https://www.sciensano.be/sites/default/files/legionella_2011-2022_nrc_rapport_english_final.pdf

Supplementary Materials Figure S1

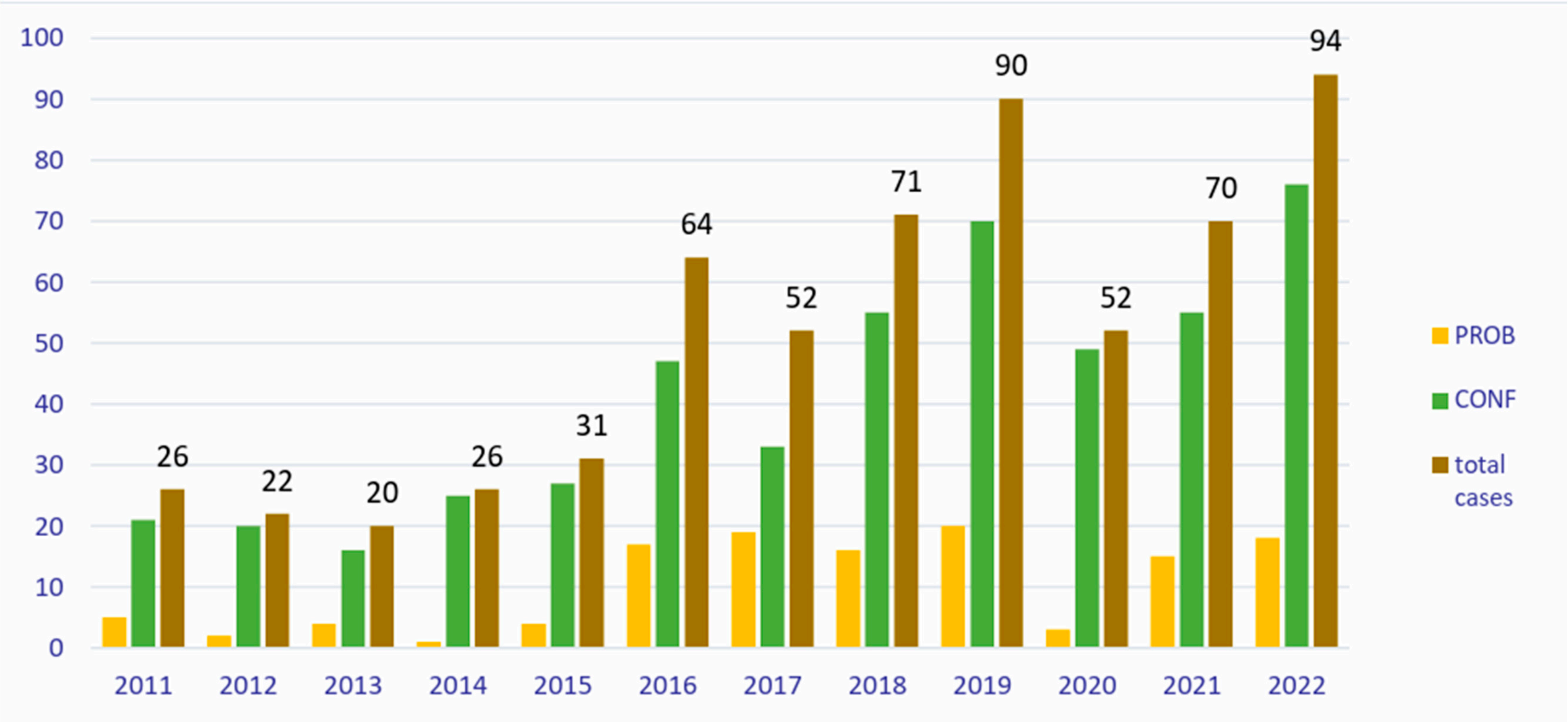

Methods Part

Supplementary Materials Table S1 - English rendition of the present survey.

Supplementary Materials Table S1

Question	***Reply***
Demographics<br>*Years of experience as a general practitioner:*	Trainee; <10 years; 10-19 years; 20-29 years; 30-40 years; >40 years.
Belgian postal code of your practice location:	Short answer required.
Practice type:	GPs Association member; Individual practice; Fee-for-service practice; Fixed-fee practice.
Practice location:	Urban; Semi-urban; Rural.
Legionnaires' Disease (LD) Experience<br>*Have you ever diagnosed a case of LD?*	Yes; No.
LD knowledge<br>*In your opinion, when are LD outbreaks most likely to occur?*	Summer; Winter.
Which patient history elements do you consider important for suspecting LD?<br>*(Select all that apply):*	Gender; Smoking history; Context of immunosuppression; Cirrhosis; Chronic pulmonary pathology; Diabetes; Return from vacations; Contact with water sources within professional setting (*e.g.* cooling tower, air conditioner); Use of swimming-pool, jacuzzi or spa.

Which symptoms and clinical findings are important for suspecting LD? (Select all that apply):	Upper airways symptoms; Flu-like syndrome; Signs of pneumonia on auscultation; Signs of acute liver failure; Abdominal pain; Generalized lymphadenopathy
Do you consider radiological confirmation important for diagnosing LD?	Yes; No.
Which diagnostic test do you prefer for LD?	*Legionella* urinary antigen test (UAT); Serology; PCR on respiratory sample; Bacterial culture on respiratory sample
Which serogroups of Legionella pneumophila are responsible for approximately 80% of LD cases?	pneumophila all serogroups; pneumophila serogroups 1,3,5 and 8; pneumophila serogroup 1; pneumophila and longbeachae.
Can a non-serogroup 1 L. pneumophila infection be detected by the UAT test?	Yes; No.
Which Legionella can most commonly be detected by available UAT kits?	pneumophila all serogroups; pneumophila serogroups 1,3,5 and 8; pneumophila serogroup 1; pneumophila and longbeachae.
What is your approach if you have a strong suspicion of LD but a negative UAT?	Short answer required.
**clinical microbiology laboratories (CML) Services** *Does your usual laboratory offer the UAT?*	Yes; No; I do not know.
If yes, when does the laboratory perform the UAT?	Always; During working hours, also on weekends; During working hours, not on weekends; I do not know.
If your laboratory doesn't offer the UAT, do you know where it can be performed?	Yes; No.

How often do you prescribe the UAT per year?	>50 times per year; Between 20 and 50 times; <20 times per year; Never.
Have you ever prescribed serological tests for suspicion of LD?	Yes; No.
How often do you prescribe serological tests for suspected LD per year?	>50 times per year; Between 20 and 50 times; <20 times per year.
Treatment *Which antibiotic do you typically prescribe for suspected LD before confirmation?*	Levofloxacin; Ciprofloxacin; Azithromycin; Clarithromycin; Amoxicillin and clavulanic acid; Doxycycline.
For how many days do you typically prescribe the initial antibiotic treatment for suspected LD?	Short answer required.
Additional information *Are you familiar with the National Reference Centre for Legionella pneumophila?*	Yes; No.
Would you be interest in having more training on LD?	Yes; No.

Results Part

Supplementary Materials Table S2 – Answers of the general practitioners about Legionnaires' disease general knowledge. Global numbers are provided for the totality of respondents, these are underlined in bold. Subsequently, general practitioners were split in six categories considering the years of experience.

Supplementary Materials Table S3 – Diagnostic tools availability for diagnosis of Legionnaire's Disease in primary care medicine. LD: Legionnaire's Disease; UAT: *Legionella* pneumophila urinary antigen test.

Supplementary Materials Table S4 - Answers of the general practitioners about Legionnaires' disease favorite treatment. Global numbers are provided for the totality of respondents, these are underlined in bold. Subsequently, general practitioners were split in six categories considering the years of experience. IQR: inter quartile range.

Supplementary Materials Table S2

**When are LD outbreaks most likely to occur? Total n= 125**	***Winter 28 (22%)***	***Summer 97 (78%)***							
Trainee n = 35	Winter 8 (23%)	Summer 27 (77%)							
Experience <10 years n = 26	Winter 4 (15%)	Summer 22 (85%)							
Experience 10-19 years n = 17	Winter 4 (23%)	Summer 13 (76%)							
Experience 20-29 years n= 13	Winter 4 (31%)	Summer 9 (69%)							
Experience 30-39 years n = 19	Winter 3 (16%)	Summer 16 (84%)							
Experience >40 years n = 15	Winter 5 (33%)	Summer 10 (67%)							
**Important elements during anamnesis Total n= 125**	**Gender 18 (14%)**	**Tobacco smoking 54 (43%)**	**Immunosuppression 86 (69%)**	**Cirrhosis 19 (15%)**	**Chronic lung disease 77 (62%)**	**Diabetes 36 (29%)**	**Return from vacations 69 (55%)**	**Water sources contact within professional setting (e.g. cooling**	**Use of swimming-pool, jacuzzi or spa 104 (83%)**

								**tower, air conditioner) 119 (95%)**	
Trainee n = 35	Gender 5 (14%)	Tobacco smoking 12 (34%)	Immunosuppression 26 (74%)	Cirrhosis 4 (11%)	Chronic lung disease 22 (63%)	Diabetes 10 (29%)	Return from vacations 24 (69%)	Water sources in professional setting 34 (97%)	Use of swimming-pool, jacuzzi or spa 27 (77%)
Experience <10 years n = 26	Gender 4 (15%)	Tobacco smoking 15 (58%)	Immunosuppression 21 (81%)	Cirrhosis 6 (23%)	Chronic lung disease 21 (81%)	Diabetes 9 (35%)	Return from vacations 18 (69%)	Water sources in professional setting 25 (96%)	Use of swimming-pool, jacuzzi or spa 21 (81%)
Experience 10-19 years n = 17	Gender 3 (18%)	Tobacco smoking 8 (47%)	Immunosuppression 14 (47%)	Cirrhosis 1 (6%)	Chronic lung disease 8 (47%)	Diabetes 6 (35%)	Return from vacations 9 (53%)	Water sources in professional setting 16 (94%)	Use of swimming-pool, jacuzzi or spa 16 (94%)
Experience 20-29 years n= 13	Gender 2 (15%)	Tobacco smoking 6 (46%)	Immunosuppression 8 (61%)	Cirrhosis 3 (23%)	Chronic lung disease 8 (61%)	Diabetes 3 (23%)	Return from vacations 4 (31%)	Water sources in professional setting 11 (85%)	Use of swimming-pool, jacuzzi or spa 10 (77%)
Experience 30-39 years n = 19	Gender 3 (16%)	Tobacco smoking 7 (37%)	Immunosuppression 8 (42%)	Cirrhosis 3 (16%)	Chronic lung disease 9 (47%)	Diabetes 6 (32%)	Return from vacations 7 (37%)	Water sources in professional setting 18 (95%)	Use of swimming-pool, jacuzzi or spa 18 (95%)

Experience >40 years n = 15	Gender 1 (7%)	Tobacco smoking 6 (40%)	Immunosuppression 9 (60%)	Cirrhosis 2 (13%)	Chronic lung disease 9 (60%)	Diabetes 2 (13%)	Return from vacations 7 (47%)	Water sources in professional setting 15 (100%)	Use of swimming-pool, jacuzzi or spa 12 (80%)
**LD symptoms, clinical findings Total n= 125**	**Upper airways symptoms *31 (25%)***	**Flu-like syndrome *113 (90%)***	**Signs of pneumonia on auscultation *115 (92%)***	**Signs of acute liver failure *21 (17%)***	**Abdominal pain *40 (32%)***	**Generalized lymphadenopathy *33 (26%)***			
Trainee n = 35	Upper airways symptoms 9 (26%)	Flu-like syndrome 32 (91%)	Signs of pneumonia on auscultation 32 (91%)	Signs of acute liver failure 5 (14%)	Abdominal pain 16 (46%)	Generalized lymphadenopathy 13 (37%)			
Experience <10 years n = 26	Upper airways symptoms 7 (27%)	Flu-like syndrome 23 (88%)	Signs of pneumonia on auscultation 25 (96%)	Signs of acute liver failure 6 (23%)	Abdominal pain 10 (38%)	Generalized lymphadenopathy 8 (31%)			
Experience 10-19 years n = 17	Upper airways symptoms 6 (35%)	Flu-like syndrome 14 (82%)	Signs of pneumonia on auscultation 16 (94%)	Signs of acute liver failure 4 (23%)	Abdominal pain 6 (35%)	Generalized lymphadenopathy 6 (35%)			
Experience 20-29 years n= 13	Upper airways symptoms 5 (38%)	Flu-like syndrome 13 (100%)	Signs of pneumonia on auscultation 11 (85%)	Signs of acute liver failure 2 (15%)	Abdominal pain 1 (8%)	Generalized lymphadenopathy 3 (15%)			

Experience 30-39 years n = 19	Upper airways symptoms 2 (10%)	Flu-like syndrome 16 (84%)	Signs of pneumonia on auscultation 18 (95%)	Signs of acute liver failure 2 (10%)	Abdominal pain 4 (21%)	Generalized lymphadenopathy 2 (10%)
Experience >40 years n = 15	Upper airways symptoms 2 (13%)	Flu-like syndrome 15 (100%)	Signs of pneumonia on auscultation 13 (87%)	Signs of acute liver failure 2 (13%)	Abdominal pain 3 (20%)	Generalized lymphadenopathy 1 (7%)
**Importance of radiological finding in LD diagnosis Total n= 125**	**Yes *94 (75%)***	**No *31 (25%)***				
Trainee n = 35	Yes *24 (67%)*	No *11 (31%)*				
Experience <10 years n = 26	Yes *17 (65%)*	No *9 (34%)*				
Experience 10-19 years n = 17	Yes *15 (88%)*	No *2 (12%)*				
Experience 20-29 years n= 13	Yes *12 (92%)*	No *1 (8%)*				
Experience 30-39 years n = 19	Yes *15 (79%)*	No *4 (21%)*				
Experience >40 years n = 15	Yes *11 (73%)*	No *4 (27%)*				
**Preferred LD diagnostic tool Total n= 125**	***Legionella* urinary antigen test (UAT) *78 (62%)***	**Serology *18 (14%)***	**PCR on respiratory sample *22 (18%)***	**Bacterial culture on respiratory sample *7 (6%)***		

Trainee n = 35	UAT<br>*27 (77%)*	Serology<br>1 (3%)	PCR on respiratory sample<br>*5 (14%)*	Bacterial culture<br>*2 (6%)*
Experience <10 years n = 26	UAT<br>*20 (78%)*	Serology<br>*2 (8%)*	PCR on respiratory sample<br>3 (11%)	Bacterial culture<br>*1 (4%)*
Experience 10-19 years n = 17	UAT<br>*10 (59%)*	Serology<br>*1 (6%)*	PCR on respiratory sample<br>3 (18%)	Bacterial culture<br>*3 (18%)*
Experience 20-29 years n= 13	UAT<br>*5 (38%)*	Serology<br>*6 (46%)*	PCR on respiratory sample<br>*2 (15%)*	Bacterial culture<br>*0 (0%)*
Experience 30-39 years n = 19	UAT<br>*10 (53%)*	Serology<br>*3 (16%)*	PCR on respiratory sample<br>*6 (32%)*	Bacterial culture<br>*0 (0%)*
Experience >40 years n = 15	UAT<br>*6 (40%)*	Serology<br>*5 (33%)*	PCR on respiratory sample<br>*3 (20%)*	Bacterial culture<br>*1 (7%)*
**Which serogroups of *Legionella* pneumophila are responsible for approximately**	**pneumophila all serogroups**<br>***46 (37%)***	**pneumophila serogroups 1,3,5 and 8**<br>***40 (32%)***	**pneumophila serogroup 1**<br>***38 (30%)***	**pneumophila and longbeachae**<br>***1 (1%)***

**80% of LD cases? Total n= 125**				
Trainee n = 35	pneumophila all serogroups *9 (26%)*	pneumophila serogroups 1,3,5 and 8 *11 (31%)*	pneumophila serogroup 1 *15 (43%)*	pneumophila and longbeachae *0 (0%)*
Experience <10 years n = 26	pneumophila all serogroups *8 (31%)*	pneumophila serogroups 1,3,5 and 8 *8 (31%)*	pneumophila serogroup 1 *10 (38%)*	pneumophila and longbeachae *0 (0%)*
Experience 10-19 years n = 17	pneumophila all serogroups *10 (59%)*	pneumophila serogroups 1,3,5 and 8 *5 (29%)*	pneumophila serogroup 1 *2 (12%)*	pneumophila and longbeachae *0 (0%)*
Experience 20-29 years n= 13	pneumophila all serogroups *8 (61%)*	pneumophila serogroups 1,3,5 and 8 *3 (23%)*	pneumophila serogroup 1 *2 (15%)*	pneumophila and longbeachae *0 (0%)*
Experience 30-39 years n = 19	pneumophila all serogroups *6 (32%)*	pneumophila serogroups 1,3,5 and 8 *8 (42%)*	pneumophila serogroup 1 *5 (26%)*	pneumophila and longbeachae *0 (0%)*
Experience >40 years n = 15	pneumophila all serogroups *5 (33%)*	pneumophila serogroups 1,3,5 and 8 *5 (33%)*	pneumophila serogroup 1 *4 (27%)*	pneumophila and longbeachae *1 (7%)*
**Can a non-serogroup 1 L. pneumophila infection be**	**Yes** ***26 (21%)***	**No** ***99 (79%)***		

**detected by the UAT test? Total n= 125**				
Trainee n = 35	Yes 6 (17%)	No 29 (83%)		
Experience <10 years n = 26	Yes 6 (23%)	No 20 (77%)		
Experience 10-19 years n = 17	Yes 5 (29%)	No 12 (71%)		
Experience 20-29 years n= 13	Yes 2 (15%)	No 11 (85%)		
Experience 30-39 years n = 19	Yes 5 (26%)	No 14 (74%)		
Experience >40 years n = 15	Yes 2 (13%)	No 13 (87%)		
**Which *Legionella* can most commonly be detected by available UAT kits? Total n= 125**	**pneumophila all serogroups *38 (30%)***	**pneumophila serogroups 1,3,5 and 8 *29 (23%)***	**pneumophila serogroup 1 *49 (39%)***	**pneumophila and longbeachae *9 (7%)***
Trainee n = 35	pneumophila all serogroups *5 (14%)*	pneumophila serogroups 1,3,5 and 8 *5 (14%)*	pneumophila serogroup 1 *23 (66%)*	pneumophila and longbeachae *2 (6%)*
Experience <10 years n = 26	pneumophila all serogroups *4 (15%)*	pneumophila serogroups 1,3,5 and 8 *7 (27%)*	pneumophila serogroup 1 *11 (42%)*	pneumophila and longbeachae *4 (15%)*

Experience 10-19 years n = 17	pneumophila all serogroups *8 (47%)*	pneumophila serogroups 1,3,5 and 8 *4 (23%)*	pneumophila serogroup 1 *3 (18%)*	pneumophila and longbeachae *2 (12%)*					
Experience 20-29 years n= 13	pneumophila all serogroups *9 (69%)*	pneumophila serogroups 1,3,5 and 8 *1 (8%)*	pneumophila serogroup 1 *3 (23%)*	pneumophila and longbeachae *0 (0%)*					
Experience 30-39 years n = 19	pneumophila all serogroups *7 (37%)*	pneumophila serogroups 1,3,5 and 8 *9 (47%)*	pneumophila serogroup 1 *2 (10%)*	pneumophila and longbeachae *1 (5%)*					
Experience >40 years n = 15	pneumophila all serogroups *5 (33%)*	pneumophila serogroups 1,3,5 and 8 *3 (20%)*	pneumophila serogroup 1 *7 (47%)*	pneumophila and longbeachae *0 (0%)*					
**If strong diagnostic suspicion, but a negative LUA, what would you do? Total n= 125**	**Contact a specialist *38 (30%)***	**Request serological testing *16 (13%)***	**Request PCR testing *15 (12%)***	**Initiate antibiotic therapy *14 (11%)***	**Request a chest X ray *14 (11%)***	**Request a culture *8 (6%)***	**Admit patient to hospital *8 (6%)***	**Request a chest CT scan *5 (4%)***	**I do not know *8 (6%)***
Trainee n = 35	Contact a specialist *10 (29%)*	Request serological testing *6 (17%)*	Request PCR testing *4 (11%)*	Initiate antibiotic therapy *5 (14%)*	Request a chest X ray *3 (9%)*	Request a culture *2 (6%)*	Admit patient to hospital *2 (6%)*	Request a chest CT scan *2 (6%)*	I do not know *2 (6%)*
Experience <10 years n = 26	Contact a specialist *8 (31%)*	Request serological testing *3 (11%)*	Request PCR testing *3 (11%)*	Initiate antibiotic therapy *2 (8%)*	Request a chest X ray *2 (8%)*	Request a culture *3 (11%)*	Admit patient to hospital *2 (8%)*	Request a chest CT scan *1 (4%)*	I do not know *2 (8%)*

Experience 10-19 years n = 17	Contact a specialist *7 (41%)*	Request serological testing *1 (6%)*	Request PCR testing *2 (12%)*	Initiate antibiotic therapy *2 (12%)*	Request a chest X ray *2 (12%)*	Request a culture *1 (6%)*	Admit patient to hospital *2 (12%)*	Request a chest CT scan *1 (6%)*	I do not know *2 (12%)*
Experience 20-29 years n= 13	Contact a specialist *5 (38%)*	Request serological testing *2 (15%)*	Request PCR testing *1 (8%)*	Initiate antibiotic therapy *1 (8%)*	Request a chest X ray *2 (15%)*	Request a culture *1 (8%)*	Admit patient to hospital *1 (8%)*	Request a chest CT scan *1 (8%)*	I do not know *0 (0%)*
Experience 30-39 years n = 19	Contact a specialist *2 (10%)*	Request serological testing *3 (16%)*	Request PCR testing *3 (16%)*	Initiate antibiotic therapy *2 (10%)*	Request a chest X ray *2 (10%)*	Request a culture *1 (5%)*	Admit patient to hospital *1 (5%)*	Request a chest CT scan *0 (0%)*	I do not know *1 (5%)*
Experience >40 years n = 15	Contact a specialist *6 (40%)*	Request serological testing *1 (7%)*	Request PCR testing *2 (13%)*	Initiate antibiotic therapy *2 (13%)*	Request a chest X ray *3 (20%)*	Request a culture *0 (0%)*	Admit patient to hospital *0 (0%)*	Request a chest CT scan *0 (0%)*	I do not know *1 (7%)*

Supplementary Materials Table S3

**Does your usual laboratory offer the UAT?**	Yes	No	I do not know
**Total n= 125**	*29 (23%)*	*2 (2%)*	*94 (75%)*

**If yes, when does the laboratory perform the UAT?**<br><br>**Total n= 29**	Always<br><br>*2 (7%)*	During working hours, also on weekends<br><br>*7 (24%)*	During working hours, not weekends<br><br>*5 (17%)*	I do not know<br><br>*15 (52%)*
**If your laboratory doesn't offer the UAT, do you know where it can be performed?**<br><br>**Total n= 96**	Yes<br><br>*24 (25%)*	No<br><br>*72 (75%)*		
**How often do you prescribe the UAT per year?**<br><br>**Total n=125**	>50 times per year<br><br>*0 (0%)*	Between 20 and 50 times<br><br>*0 (0%)*	<20 times per year<br><br>*22 (18%)*	Never<br><br>*103 (82%)*
**Have you ever prescribed serological tests for suspicion of LD?**<br><br>**Total n=125**	Yes<br><br>*27 (22%)*	No<br><br>*98 (78%)*		
**How often do you prescribe serological tests for suspected LD per year?**<br><br>**Total n=27**	>50 times per year<br><br>*0 (0%)*	Between 20 and 50 times<br><br>*0 (0%)*	<20 times per year<br><br>*27 (100%)*	

Supplementary Materials Table S4

Preferred antibiotic if suspected LD	Levofloxacin	Ciprofloxacin	Azithromycin	Clarithromycin	Doxycycline	Amoxicillin and clavulanic acid	Median days of treatment and interquartile range (IQR)
Total n= 125	***29 (23%)***	***12 (10%)***	***38 (31%)***	***30 (24%)***	***10 (8%)***	***6 (5%)***	***10 (IQR: 7-14)***
Trainee n = 35	6 (17%)	3 (9%)	12 (34%)	7 (20%)	4 (11%)	3 (9%)	7 (IQR: 5-10)
Experience <10 years n = 26	7 (27%)	3 (11%)	7 (27%)	7 (27%)	2 (8%)	0 (0%)	8 (IQR: 7-10)
Experience 10-19 years n = 17	5 (29%)	1 (6%)	4 (23%)	5 (29%)	1 (6%)	1 (6%)	10 (IQR: 10-17)
Experience 20-29 years n= 13	6 (46%)	2 (15%)	2 (15%)	1 (8%)	0 (0%)	2 (15%)	10 (IQR: 7-14)
Experience 30-39 years n = 19	3 (16%)	2 (10%)	6 (32%)	5 (26%)	3 (16%)	0 (0%)	10 (IQR: 6-15)
Experience >40 years n = 15	2 (13%)	1 (6%)	7 (47%)	5 (33%)	0 (0%)	0 (0%)	10 (IQR: 7-15)
